# Unusual Fungal Infections in Renal Transplant Recipients

**DOI:** 10.1155/2015/292307

**Published:** 2015-02-26

**Authors:** Mahesh Eswarappa, P. Vijay Varma, Rakesh Madhyastha, Sujeeth Reddy, M. S. Gireesh, K. C. Gurudev, Vijaya V. Mysorekar, Beena Hemanth

**Affiliations:** ^1^Department of Nephrology, M. S. Ramaiah Medical College and Teaching Hospital, MSR Nagar, MSRIT Post, Bangalore, Karnataka 560054, India; ^2^Department of Pathology, M. S. Ramaiah Medical College and Teaching Hospital, MSR Nagar, MSRIT Post, Bangalore, Karnataka 560054, India; ^3^Department of Microbiology, M. S. Ramaiah Medical College and Teaching Hospital, MSR Nagar, MSRIT Post, Bangalore, Karnataka 560054, India

## Abstract

Fungal infections are an important cause of morbidity and mortality in renal transplant recipients. The causative agent and the risk factors differ depending on the period after the kidney transplant. Also the incidence varies according to the geographical area. We are reporting three cases of fungal infections in renal transplant recipients. Two of them have etiological agents which are common among immunosuppressed patients, but with an atypical clinical presentation, while one of them is a subcutaneous infection caused by a less frequent dematiaceous fungus, *Aureobasidium pullulans*. These cases highlight how a high index of clinical suspicion and prompt diagnosis is very much essential for better outcome. The emerging fungal infections and paucity of data regarding their management pose a challenge to the transplant physicians.

## 1. Introduction

Fungal infections account for 5% of all infections in renal transplant recipients  [1]. The incidence varies according to the geographical area. Because of environmental exposure and the effects of immunosuppressive regimens, systemic mycosis is a significant problem in transplant patients worldwide and remains the major cause of death in those individuals [2]. In an Indian study 6.1% of renal transplant recipients were affected by systemic fungal infections and resulted in a 63% mortality rate [3]. The causative agent and the risk factors differ depending on the period after the kidney transplant.* Aspergillus* species, Mucorales species,* Candida* species, and* Cryptococcus neoformans* are the opportunistic fungi that cause most infections.

Herein we report three cases of fungal infection in renal transplant recipients. Two of them have etiological agents which are common among immunosuppressed patients, but with an atypical clinical presentation, while one of them is a subcutaneous infection caused by a less frequent dematiaceous fungus,* Aureobasidium pullulans*.

## 2. Case 1

A 33-year-old male who underwent renal transplantation in May 2012 with his mother as donor (haplomatch) was started on antituberculous treatment (ATT) for pulmonary tuberculosis prior to transplant from December 2011 and continued up to September 2012. In view of graft dysfunction biopsy was done in November 2013 which showed evidence of interstitial fibrosis and tubular atrophy (IF/TA). This patient presented to us in April 2014 with pain in the posterior aspect of the right side of the chest. He was on tacrolimus [(TAC) 3 mg/day], mycophenolate mofetil [(MMF) 1 g/day], and prednisolone (10 mg/day) immunosuppression. Serum creatinine at the time of admission was 2.5 mg/dL.

**Figure 1:**
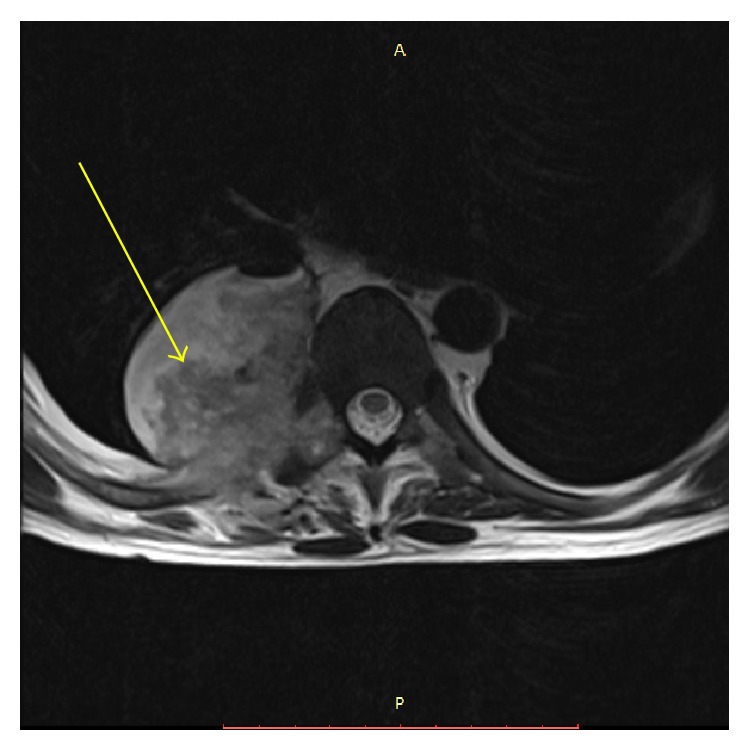
MRI spine (axial T1 and T2) showing paraspinal abscess on the right side with an air fluid level (arrow).

Chest radiograph showed homogenous lobulated opacity in the right hilar region. Computed tomography (CT) of the chest revealed a cold abscess in the D9 vertebra-rib region. In the background of old pulmonary tuberculosis he was empirically restarted on ATT. However patient had worsening of symptoms with fever spikes. MRI spine revealed well-defined heterogeneously hyperintense signal lesion in the paravertebral region bilaterally and predominantly on the right side (Figure 1). Surgical drainage of the lesion was done and pus showed fungal elements of* Aspergillus* species. He was treated with IV liposomal amphotericin B [(LAmpB) 3 mg/kg/day] for 7 days followed by oral voriconazole (VOR) 200 mg twice a day and the dose of TAC was adjusted accordingly. Patient developed progressive worsening of renal function and severe metabolic acidosis requiring initiation of hemodialysis. He was also treated with broad spectrum antibiotics. On day 14 of admission, patient developed acute onset paraplegia and hypotension requiring inotropic support. Repeat MRI showed collapse of T8, T9 vertebrae with evidence of spinal cord indentation. However he could not be taken for surgical intervention owing to poor hemodynamic status. Patient developed refractory shock and ultimately succumbed.

## 3. Case 2

**Figure 2:**
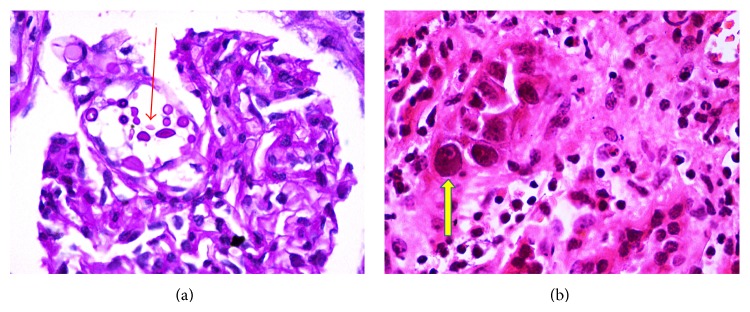
Glomerulus exhibits intracapillary spherical fungal structures that show PAS positive capsules consistent with cryptococcal organism (red arrow). Few of the tubular epithelial cells show homogenous smudgy appearing intranuclear inclusions (yellow arrow).

A 34-year-old lady underwent renal transplantation in May 2012 with her brother as donor (HLA match—NIL). She was on triple immunosuppressive regimen. In view of graft dysfunction (serum creatinine: 2.5 mg/dL), graft biopsy was done in October 2013 which showed interstitial fibrosis and tubular atrophy (IF/TA). This lady presented to us with complaints of fever and burning micturition in December 2014.* E. coli* was isolated from the urine culture and she was started on appropriate IV antibiotics. However she continued to have fever spikes in spite of antibiotic therapy. Further evaluation revealed* Cytomegalovirus* (CMV) infection and she was started on intravenous ganciclovir (GCV) 250 mg/day. On day 7 of initiation of therapy patient developed leucopenia for which MMF dose was tapered and later stopped and intravenous GCV was withheld. In the meanwhile patient had severe dyspeptic symptoms for which esophagogastroduodenal endoscopy was done which showed esophageal candidiasis. She was started on intravenous fluconazole 100 mg/day and TAC dose was adjusted accordingly. Patient developed progressive graft dysfunction (serum creatinine: 6.5 mg/dL) for which she was initiated on hemodialysis and later subjected to graft biopsy which showed intracapillary spherical fungal structures within the glomerulus that showed periodic acid-Schiff stain (PAS) positive capsules consistent with cryptococcal organisms. Also tubular epithelium showed features of CMV nephritis (Figure 2). She was restarted on intravenous GCV, once the leucopenia resolved. In view of multiple coinfections (CMV nephritis, cryptococcal infection, and esophageal candidiasis) patient was given the option of withdrawal of immunosuppression in order to prevent dissemination. However consent for the withdrawal of immunosuppression was not given and she was started on intravenous LAmpB 150 mg/day. In spite of 10-day treatment with LAmpB there was no improvement in graft function and she was dialysis-dependent. Patient ultimately succumbed to the underlying illness.

## 4. Case 3

**Figure 3:**
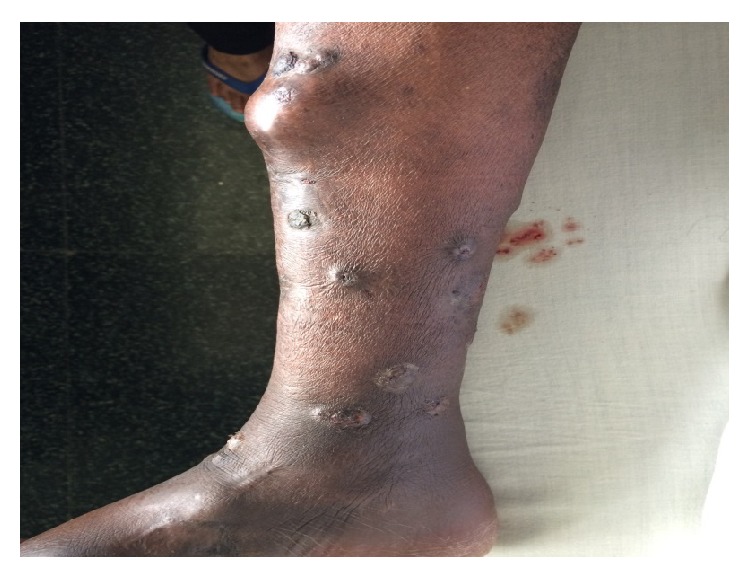
Photograph showing emerging subcutaneous nodule with healed lesions over the right lower limb.

A 56-year-old male, a case of end stage renal disease due to diabetic nephropathy, underwent renal transplantation in June 2011, donor being his sister (HLA match—full house match). He was on triple immunosuppressive regimen. In June 2013 he presented to us with a nonhealing wound over the sole of right foot following a trauma and multiple subcutaneous nodules over the right leg (Figure 3). He was subjected to biopsy of the ulcer edge and pus from the nodules was sent for bacterial and fungal culture sensitivity.

He was empirically started on IV antibiotics, pending further reports. Pus from the subcutaneous nodules showed fungal elements. He was started on oral itraconazole (ITR) 200 mg/day. The serum TAC levels increased to 23 ng/mL after one-week treatment with ITR and the TAC dose was reduced to a mere 0.5 mg/day. Growth was observed on Sabouraud medium on day 10 of incubation which was later reported to be due to a mould* Aureobasidium pullulans*. He was continued on oral ITR 200 mg/day and the skin lesions gradually disappeared. Two months later patient developed recurrence of lesions while still being on ITR.

Hence treatment was changed over to oral VOR 200 mg/day and the TAC dose was adjusted accordingly. Lesions gradually disappeared over a period of 1 month. Treatment with VOR was continued for another 2 months following the resolution and then stopped.

## 5. Discussion

Fungal infections among renal transplant recipients are an important cause of mortality. The symptoms of systemic fungal infections are nonspecific, particularly in their early stages [2]. In the first two cases described above, although the infections were caused by common fungal agents among the transplant recipients, the site of involvement was very unusual. The third case of subcutaneous mycosis due to* Aureobasidium pullulans* is a rare infection with only a few cases reported so far.

In the first case, the diagnosis was delayed due to initial treatment with ATT as the patient had prior pulmonary tuberculosis and also because tuberculous paraspinal abscess is a more common entity in tropical countries like India. The frequency of invasive aspergillosis (IA), the second-highest cause of invasive fungal infection in renal transplant recipients after candidiasis, ranges from 0.5% to 2.2%, with a mortality rate up to 88% [4–7]. Although pulmonary involvement is the most common presentation of IA, the spectrum of disease is broad and virtually every organ of the body has been reported to be affected [8]. Spinal cord involvement by* Aspergillus* is a rare entity and could be in the form of paraspinal abscess, epidural abscess, vertebral osteomyelitis, discitis, or spinal cord necrosis [9]. A similar case scenario of* Aspergillus* paraspinal abscess causing paraparesis and ultimately death due to fungal sepsis has been described [10]. This case highlights how misdiagnosis at an early stage of a serious infection in a transplant recipient can prove to be fatal. A high index of clinical suspicion is hence necessary.

The second case had multiple coinfections: CMV nephritis, esophageal candidiasis, and cryptococcosis of the allograft. The increased occurrence of fungal infections in the background of immunomodulatory viruses such as* Cytomegalovirus* is well known. Majority of cases (53–72%) of cryptococcal disease among solid organ transplant (SOT) recipients are disseminated or involve the CNS [11]. Overall, 61% of the SOT recipients in one report had disseminated disease, 54% had pulmonary disease, and 8.1% had skin, soft-tissue, or osteoarticular cryptococcosis [12]. The detection of cryptococcal elements in the renal allograft has been rarely described in the literature. In one French study of 11 SOT recipients, 3 had urinary tract infection [13]. The case highlights the need to save the life first and then to save the kidney when inevitable.

The third case of subcutaneous nodular fungal infection caused by* Aureobasidium pullulans* is the first case being reported from India.* Aureobasidium pullulans* is a saprophytic dematiaceous fungus widely distributed in the environment, and it can be isolated from soil, decaying plant debris, wood, rock, and household dust as well as human hair, skin, and nails [14–16].

It is the best known* Aureobasidium* species for causing emerging human diseases including peritonitis in patients on peritoneal dialysis, splenic abscess, meningitis, skin, and soft tissue infections, as well as septicemia in patients with malignancies or receiving major surgery [16].

Franco et al. described a case of chromomycosis in a renal transplant recipient caused by* A. pullulans* following penetrating trauma in the same skin area with the presentation as an ulcerative lesion resembling squamous cell carcinoma [17]. In the present case also patient had a wound over the sole of the right foot prior to the development of the skin lesions which probably could have been the source of infection.

Arranz Sánchez et al. reported similar [18] skin lesions in a renal transplant recipient caused by* A. pullulans* which subsided within 2 months of ITR therapy with no relapse at one year of follow-up. However in our case the lesions recurred after 2 months despite the patient being on antifungal agents. How long these lesions have to be treated has not been clearly defined [18, 19]. This case also highlights how crucial it is to optimize the TAC dose and monitor for nephrotoxicity when the patient is treated with antifungal drugs such as triazoles, owing to the drug interactions.

## 6. Conclusion

The present case series highlights the unusual fungal infections and their manifestations in the renal transplant population. A high index of clinical suspicion and prompt diagnosis is very much essential for better outcome. In certain clinical situations, the decision to withdraw the immunosuppression and forgo the graft might be the call of the hour. The emerging fungal infections and paucity of data regarding their management pose a challenge to the transplant physicians.
